# Elevated MTA1 induced the migration and invasion of renal cell carcinoma through the NF-κB pathway

**DOI:** 10.1186/s12894-020-00731-1

**Published:** 2020-10-15

**Authors:** Cai Lv, Yuan Huang, Qingqing Lei, Zhenxiang Liu, Shixing Shen, Wenxia Si

**Affiliations:** 1 Department of Urology, Haikou Municipal Hospital, Haikou, 570208 Hainan China; 2 Department of Neurology, Haikou Municipal Hospital, Haikou, 570208 Hainan China; 3 Department of Urology, Danzhou People’s Hospital, Danzhou, 571799 Hainan China; 4 Hubei Key Laboratory for Kidney Disease Pathogenesis and Intervention, Huangshi Central Hospital of Edong Healthcare Group, Hubei Polytechnic University School of Medicine, Huangshi, China

**Keywords:** MTA1, RCC, Cell migration and invasion, NF-κB, MMP2/MMP9, E-cadherin

## Abstract

**Background:**

The metastasis-associated gene 1 (MTA1) has been extensively reported as a crucial oncogene, and its abnormal expression has been associated with the progression of numerous cancers. However, the role of MTA1 in renal cell carcinoma (RCC) progression and metastasis remains unclear. Herein, we investigated the expression of MTA1 and its role in RCC.

**Methods:**

109 matched clear cell RCCs (ccRCCs) and corresponding normal tissue samples were analyzed via immunohistochemistry to test the expression of MTA1. Human A498 cell lines were transfected with pcDNA3.1-Flag (control) or Flag-MTA1 to overexpress MTA1 or with specific interfering RNA (si-MTA1) or specific interfering negative control to knockdown MTA1 expression. Transfected cells were used in wound healing and transwell invasion assay. Quantitative real time polymerase chain reaction was used to assess the effect of MTA1 on MMP2/MMP9 and E-cadherin gene expression. Western blot was used to qualify the phosphorylation of p65.

**Results:**

Herein, we found a significantly increased expression of MTA1 in 109 ccRCCs, compared to the corresponding normal tissue. In addition, the overexpression of MTA1 in A498 cells facilitated cell migration and invasion, while the down-regulation of MTA1 expression using specific interfering RNA sequences could decrease cell migration and invasion. Furthermore, we showed that MTA1 is up-regulated in ccRCCs, which contributes to the migration and invasion of human kidney cancer cells by mediating the expression of MMP2 and MMP9 through the NF-κB signaling pathway. Similarly, we found that MTA1 could regulate E-cadherin expression in RCCs.

**Conclusions:**

MTA1 is overexpressed in RCC and is involved in the progression of RCC through NF-κB.

## Background

Renal cell carcinoma (RCC) is annually increasing worldwide, often with the lack of early-warning signs, as only up to 10% of RCC patients present characteristic clinical symptoms [1]. Kidney cancer is a highly heterogeneous malignancy, and clear cell RCC (ccRCC) is the most common pathologic type, accounting for 75% of all renal malignancies [2]. The five-year survival rates of RCC patients with stage I, II, III, and IV renal cancer are reported to be 94.7%, 88.9%, 68.8%, and 19.3%, respectively [3]. Thus, earlier detection of RCC places a greater emphasis on the preservation of kidney function and treatment of RCC. With the understanding of the molecular mechanisms underlying RCCs, targeted therapies with small molecules, such as sorafenib and sunitinib, have been effective in treating RCCs [4]. Therefore, further understanding of the underlying mechanisms of RCC development and search for new therapeutic targets dependent on these mechanisms are needed.

The metastasis-associated gene 1 (MTA1) is a crucial gene involved in cancer metastasis and is overexpressed in many cancers, including gastric, ovarian, prostate, and breast cancer [5–9]. MTA1 has been reported as a key component of the nuclear remodeling and deacetylation complex, which regulates metastasis-associated gene expression, including cell migration and invasion [10]. Moreover, MTA1 could mediate the migration and invasion of cancers by regulating the phosphorylation of various intracellular proteins involved in signaling pathways, such as AKT, hypoxia, and hedgehog signaling [7, 8, 11]. MTA1 has been reported to promote cancer cell invasion by depressing the E-cadherin expression [12, 13]. Recently, MTA1 has been recognized as a prognostic biomarker in lung cancer and is associated with poor prognosis in these patients [5]. However, the role of MTA1 in kidney cancer and its molecular mechanisms are currently unknown.

This study aimed to examine the relationship between MTA1 and RCC as well as the MTA1 expression and prognosis of RCC patients. Based on the clinical results, we further performed in vitro experiments to investigate the effects of MTA1 expression on the metastasis of RCC cell line and its molecular mechanisms.

## Methods

### Patients and tissue samples

The 109 matched ccRCC and adjacent normal tissue specimens were obtained from the Haikou Municipal Hospital (Haikou, China).This study was approved by the Ethics Committee of Haikou Municipal Hospital and by Hubei Polytechnic University. Written informed consent was obtained from all patients.

### Cell culture and reagents

The human kidney cancer cell line A498 was purchased from the China Center for Type Culture Collection (Wuhan, China). Cells were cultured in DMEM (Hyclone) with 10% FBS (GIBCO) and incubated in a cell incubator at 37 °C and 5% CO_2_.

A498 cells were transfected with Flag-MTA1, specific interfering RNA (siRNA), and their respective controls pcDNA3.1-Flag and siNC using Lipo2000 (Invitrogen). MTA1 siRNA (si-MTA1) and siNC were purchased from Ribobio (Guangzhou, China). The NF-κB inhibitor, pyrrolidine dithiocarbamate (PDTC), was purchased from MedChemEepress.

### Immunohistochemistry

The ccRCC and adjacent tissues were treated following our previous report [14]. Briefly, tissues were fixed with formalin (Beijing Suolebao Biotechnology), sectioned, deparaffinized, and hydrated following endogenous peroxidase inactivation and antigen recovery. Sections ware incubated with MTA1 antibody (14682–1-AP, Proteintech) at 4 °C overnight, followed by the secondary antibody (GB23303; Servicebio) at 37 °C for 30 min. After washing, the sections were then treated with diaminobenzidine reagent (DAB horseradish peroxidase color development kit; Beyotime) and visualized at 200× and 400× magnification with a light microscope (Olympus) in a double-blind analysis by two pathologists. For each specimen, the number of positive cells was randomly counted and was scored based on the average percentage of positive cells in five high-power fields (≤ 5%, 0 points; 6–25%, 1 point; 26–50%, 2 points; 51–75%, 3 points; and > 75%, 4 points). In addition, the specimens were scored according to the intensity of staining: no staining, 0 points; light yellow, 1 point; brown, 2 points; light brown, 3 points; and dark brown, 4 points. Finally, the two scores were added and categorized as follows: 0–2, negative; 3–5, weak positive; and 6–8, strong positive. To facilitate statistical analysis, a score of ≥ 3, points was classified as positive.

### Quantitative real time polymerase chain reaction

Total RNA was isolated using the TRIzol kit (Takara) as previously described [15]. A total of 2 µg RNA was used to transcribe cDNA using reverse transcription kit (Promega). Quantitative real time polymerase chain reaction (qRT-PCR) was performed using ABI QuantStudio version 5 (Applied Biosystems; Thermo Fisher Scientific, Inc.). The results were analyzed using the 2^−ΔΔCt^ method and GAPDH as an internal reference.

The primers used in the study were as follows:

**Table:** 

GAPDH-F	ATCGTGGAAGGACTCATGACC
GAPDH-R	AGGGATGATGTTCTGGAGAGC
MMP2-F	CCCACTGCGGTTTTCTCGAAT
MMP2-R	CAAAGGGGTATCCATCGCCAT
MMP9-F	AGACCTGGGCAGATTCCAAAC
MMP9-R	CGGCAAGTCTTCCGAGTAGT
E-cadherin-F	CGAGAGCTACACGTTCACGG
E-cadherin-R	GGGTGTCGAGGGAAAAATAGG

### Western blot assay

Western blot assay was performed as previously described [16]. In brief, the treated A498 cells were lysed with RIPA lysis buffer containing proteinase inhibitors and phosphatase inhibitors (Roche). Then, the obtained cell lysates were used for western blot analysis. The MTA1 (BA2749) was from BOSTER, and β-actin (10230-1-AP) antibodie was purchased from Proteintech, and p65 (AF5006) and p-p65 (AF2006) antibodies were obtained from Affinity Biosciences.

### Cell migration assay: wound healing

Cultured A498 cells (at 80% confluence) were transfected with Flag-MTA1, si-MTA1, and their relative control vectors pcDNA3.1-Flag and siNC using Lipo2000 (Invitrogen). After 12 h, a wound (scratch) was artificially created using the tip of a 10 µl pipette in the center of middle well containing the adherent cells. The width of the scratch was regularly measured under 100 × magnification to measure the gap closure rate as an indicator of cell migration data on wound healing (Olympus microscope).

### Cell invasion assay

A498 cells were transfected with Flag-MTA1, pcDNA3.1-Flag, si-MTA1, and siNC as described above. After 24 h, cells from each condition were transferred in to the upper part of matrigel-coated (rehydrated with medium, 1:8) transwell chambers with 200 µL serum-free DMEM, while the lower part of the chamber contained 500 µL DMEM with 10% FBS. After culturing for 12 h in the incubator, the invaded cells on the outer surface of the filter screen were fixed with methyl alcohol for 10 min and were stained by 0.1% crystal violet for 10 min; then, they were photographed using an Olympus light microscope using a 40 × objective and counted using Image J.

### Statistical analysis

All statistical analyses were performed using SPSS version 17.1. The correlations between the MTA1 expression and the clinical-pathological characteristics were determined using the χ^2^ test. Data are expressed as the mean ± standard deviation. Statistical significance was analyzed using the Student’s *t*-test between the two groups and ANOVA for multiple groups. A *p *value < 0.05 was considered significant.

## Results

### MTA1 was highly expressed in RCC cells and tissues

**Fig. 1:**
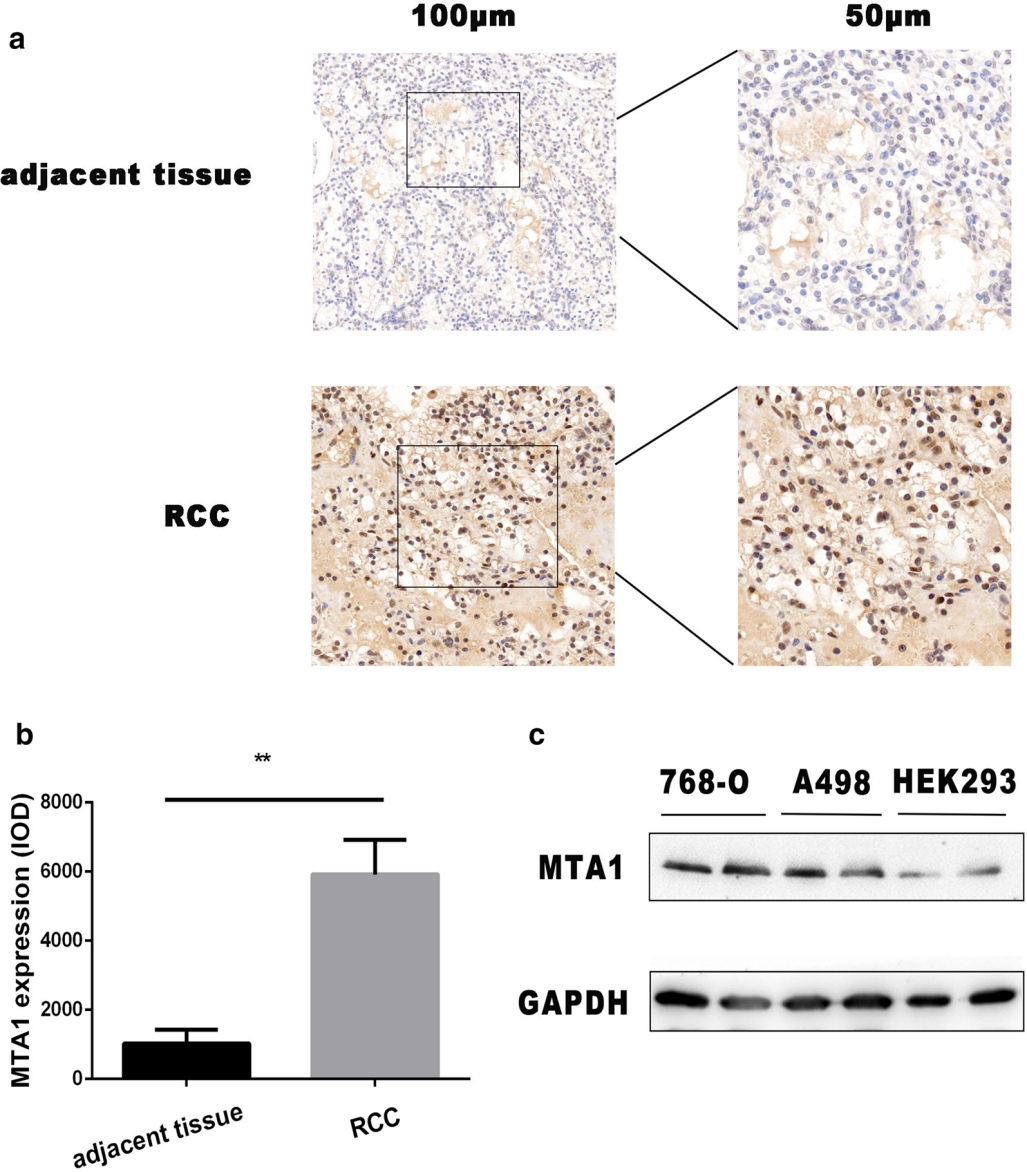
The expression of MTA1 is up-regulated in RCCs. **a** Immunohistochemistry was used to characterize the expression of MTA1 in RCC and adjacent tissues. MTA1 was highly expressed in ccRCCs, compared to very weak staining in adjacent tissue. **b** Image Pro Plus was used for the statistical analysis of the positive signal (IOD) of MTA1 expression in ccRCCs and the adjacent tissue. The expression of MTA1 was significantly up-regulated in ccRCCs. **p < 0.01; scale bar: 100 µm and 50 µm. **c** The expression of MTA1 in RCC cell lines. Normal renal cell line HEK293T and RCC cell lines A498 and 768-O cell lysates were used in western blotting analysis with MTA1 and GAPDH antibodies. The bands of MTA1 were from Additional file 1: Fig. 1 and gapdh were from Additional file 1: Fig. 1

To explore the relationship between the MTA1 expression and RCC progression, the expression of MTA1 in 109 pairs of ccRCCs and adjacent tissues was analyzed by immunohistochemistry. As shown in Fig. 1a, b, MTA1 was highly expressed in ccRCCs, while it was poorly expressed in adjacent tissues. In addition, we compared MTA1 expression in corresponding normal tissues and in ccRCC cell lines. In A498 and 768-O RCC cell lines, the MTA1 expressions were higher than those of normal HEK293T cells (Fig. 1c). Further analysis of 109 ccRCCs and adjacent tissue pairs showed that 74.3% (81/109) of ccRCCs had a positive expression of MTA1 (Table 1). As shown in Table 2, the MTA1 expression was associated with the age, tumor (T) stage, and grade in ccRCC patients (*p* < 0.05; Table 2). These clinical data suggest MTA1 may play a crucial role in RCC progression.

**Table 1:** MTA1 expression in 109 pairs of RCC and adjacent tissues

Tissue type	MTA1		χ^2^ value	*p* value
	Positive	Negative		
Renal cell carcinoma	81	28	44.114	0.000
Adjacent tissue	32	77		

Note: MTA1, metastasis-associated gene 1; RCC, renal cell carcinoma

**Table 2:** Relationship between MTA1 expression and clinical-pathological data in ccRCC patients

Clinical-pathological feature	MTA1		χ^2^ value	*p* value
	Positive, n	Negative, n		
Age, year				
< 60	29	16	3.909	0.048*
≥ 60	52	12		
Sex				
Male	56	16	1.335	0.248
Female	25	12		
T stage				
T1	18	13	6.159	0.046*
T2	38	10		
T3	25	5		
Grade				
G1	20	14	6.813	0.033*
G2	27	8		
G3	34	6		
Lymph node metastasis				
With	19	4	1.051	0.305
Without	62	24		

*p < 0.05

### MTA1 promotes the migration of RCC cells

**Fig. 2:**
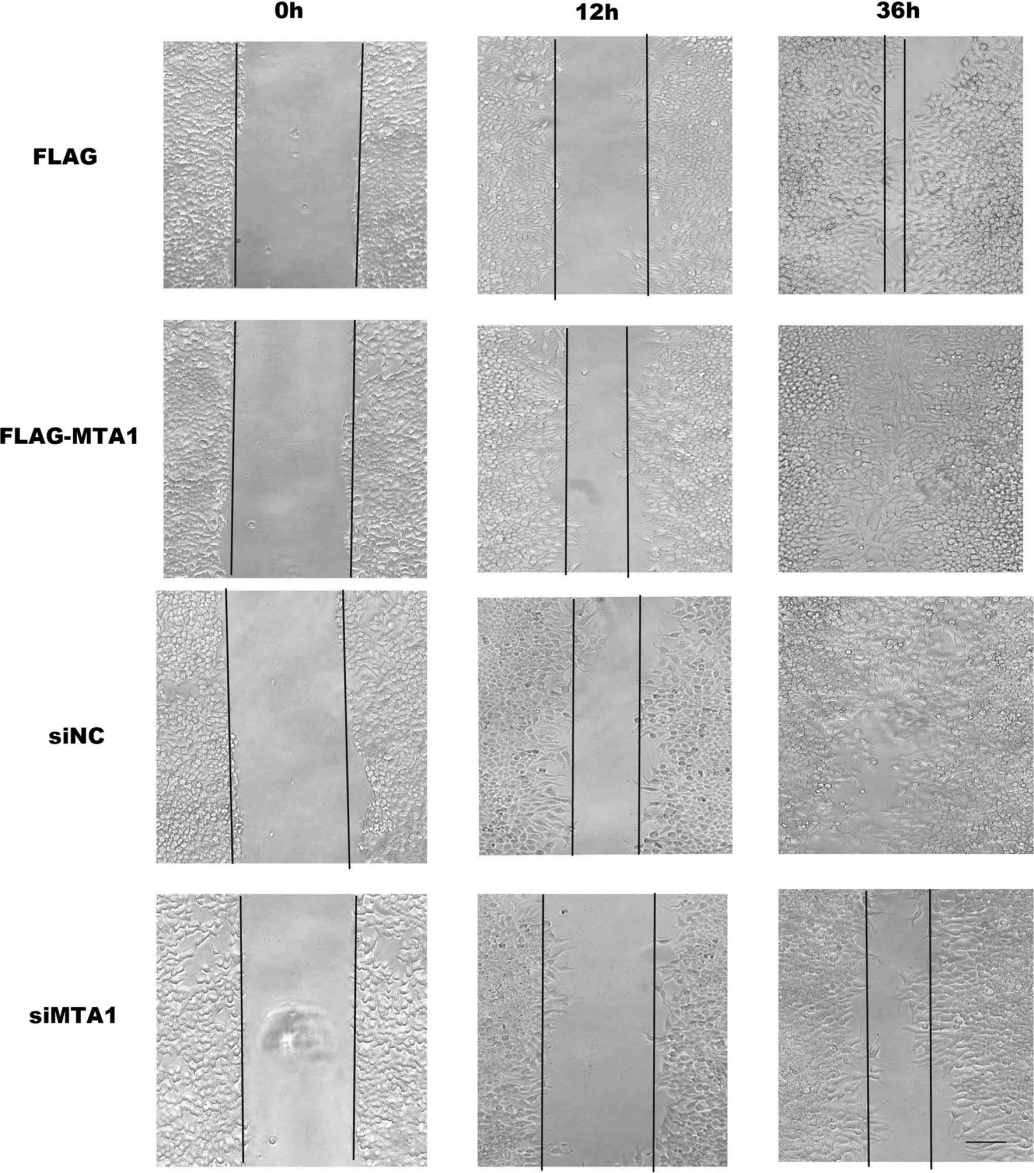
MTA1 promotes A498 cell migration. A498 cells were transfected with pcDNA3.1-Flag, Flag-MTA1, siNC, and si-MTA1. After 24 h, cells in the four conditions were subjected to the wound healing assay. Macrographs were taken under ×100 magnification

As MTA1 plays an important role in migration and invasion, we explored its effect on the migration of RCC cells. A498 cells were engineered to overexpress MTA1 following transfection with Flag-MTA1 or MTA1 knockdown with si-MTA1 and the respective control vectors pcDNA3.1-Flag and negative control (siNC). After 48 h, cells were harvested for WB to test the MTA1 protein levels. As shown in Fig. 4a, the expression of MTA1 was markedly overexpressed in control conditions or knocked-down when transfected with Flag-MTA1 or si-MTA1. For wound healing assay using A498 cells, similar transfections were performed using pcDNA3.1-Flag, Flag-MTA1, Flag-MTA1, siNC, and si-MTA1. After 12 h of transfection, cells were subjected to the wound healing assay for 12 h and 36 h. The wound healing assays showed that overexpression of MTA1 significantly increased migration of A498 cells (Fig. 2; lines 1, 2), whereas the knockdown of MTA1 expression decreased migration of A498 cells (Fig. 2; lines 3, 4).

### MTA1 supports A498 cell invasion

**Fig. 3:**
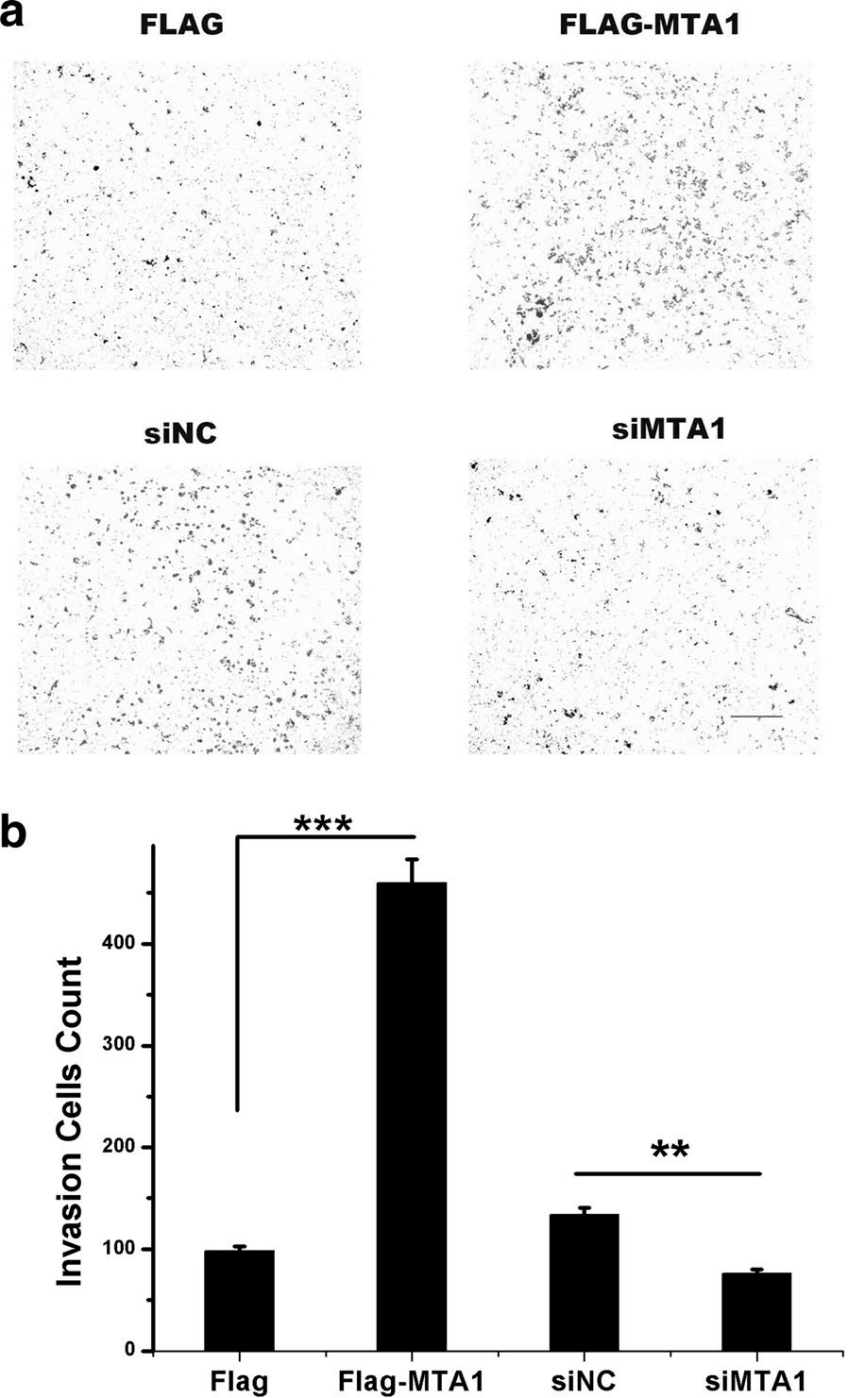
MTA1 enhances the invasion of A498 cells. **a** A498 cells were transfected with pcDNA3.1-Flag, Flag-MTA1, siNC, and si-MTA1. After 24 h, the transwell cell invasion assay using A498 cells was performed, and macrographs were taken under ×40 magnification. Scale bar: 50 µm. **b** Image J was used to count the transmigrated cells and Student’s *t* test to analyze differences. **p < 0.01; ***p < 0.001

To identify whether MTA1 has an effect on the invasion of RCC cells, A498 cells were again transfected with Flag-MAT1, si-MTA, and the negative controls (pcDNA3.1-Flag and siNC) as indicated above. After 36 h of transfection, cells were used in transwell matrigel invasion assays (in vitro). The results are shown in Fig. 3. Compared to the negative control, the overexpression of MTA1 increased the invasion of A498 cells. While the knockdown of the MTA1 expression reduced the A498 cell invasion, compared to the siNC condition.

### *MTA1 regulates the NF-*κ*B signaling pathway and MPP2/MMP9 as well as the E-cadherin gene expression*

**Fig. 4:**
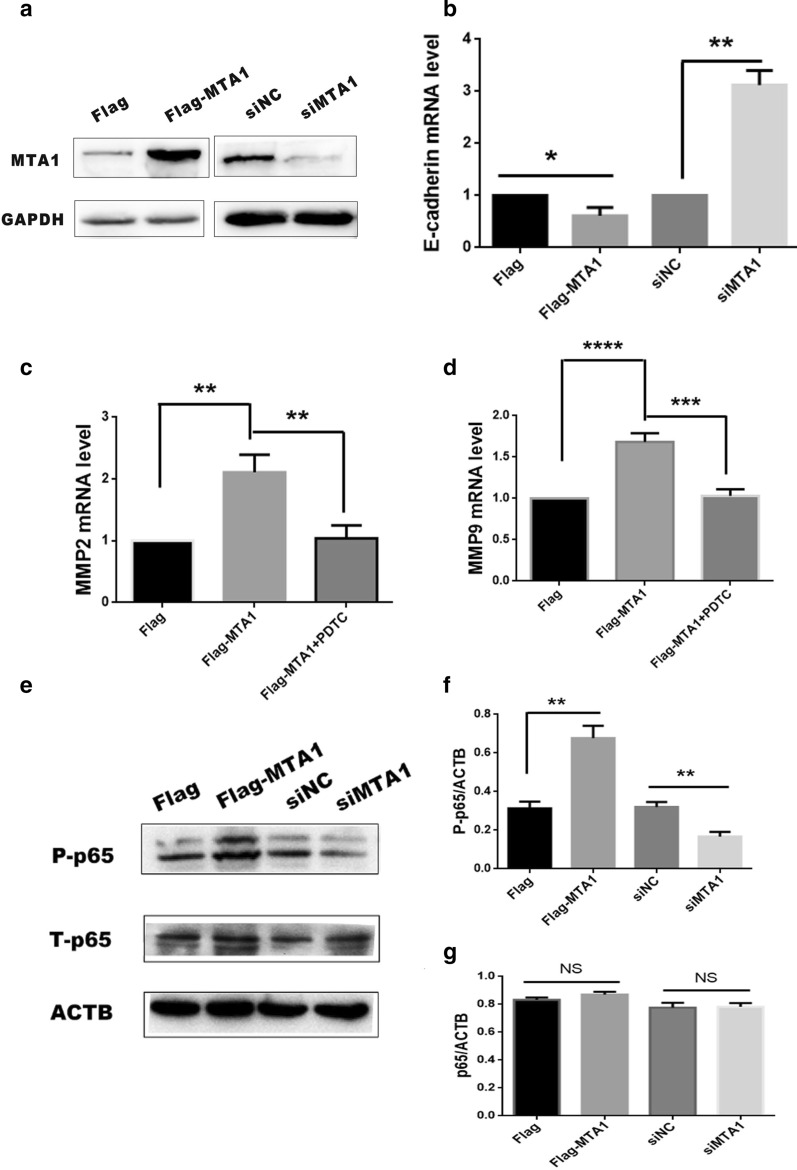
MTA1 regulated the expression of metastasis-related factors via the NFκB pathway. A498 cells were transfected with pcDNA3.1-Flag, Flag-MTA1, siNC, and si-MTA1. **a** Protein levels of MTA1; cells were collected for WB with MTA1 and GAPDH antibodies. The bands of over-expressed MTA1 were from Additional file 1: Fig. 2A and down-expressed MTA1 were from Additional file 1: Fig. 2B with arrow. **b** Cells were lysed and used in qRT-PCR assays to measure the E-cadherin mRNA expression. Statistical analysis was performed using Student’s *t* test. **b**, **c** A498 cells were transfected with pcDNA3.1-Flag and Flag-MTA1. After 36 h, the Flag-MTA1 group was treated with 10 nM PDTC for 12 h. Then cells were harvested for qRT-PCR evaluation to measure the mRNA expression of MMP2 (**b**) and MMP9 (**c**). Statistical analysis was performed using ANOVA. **d** A498 cells were transfected with pcDNA3.1-Flag, Flag-MTA1, siNC, and si-MTA1. After 48 h, cells were subjected to western blotting analysis using antibodies targeting p-p65, p65 and ACTB. The bands of p-p65, p65 and ACTB were from Additional file 1: Fig. 3. **e, f** Image J was used to calculate the gray scanned bands of p-p65 (**b**) and p65 (C). *p < 0.05; **p < 0.01; ***p < 0.001, ****p < 0.0001; *NS * not significant

Metalloproteinase have been reported to stimulate tumor cell invasion and migration [11]. Furthermore, MTA1 was found to promote invasion by depressing the transcriptional expression of E-cadherin [17]. Thus, we verified the expression of E-cadherin, MPP2, and MPP9 in the MTA1 overexpressed and knocked-down A498 cells by qRT-PCR. The results showed that MTA1 could repress the expression of E-cadherin (Fig. 4a) and up-regulate the expression of MPP2 and MPP9 (Fig. 4b, c).

As is well known, the NF-κB pathway is involved with the regulation of MPP expression [18–20]. To further investigate the mechanism of MTA1-regulated expression of MPP2 and MPP9, western blotting analysis was used to test the NF-κB signaling pathway. As shown in Fig. 4d–f, MTA1 overexpression decreased the phosphorylation of p65 (p-p65), with no change in the levels of total p65. When we used the NF-κB inhibitor, PDTC (10 nM), to block the NF-κB signaling pathway, MTA1 had no effect on the expression of MPPs (Fig. 4b, c). These results suggested that MTA1 might regulate the expression of MPP2 and MPP9 through NF-κB.

**Fig. 5:**
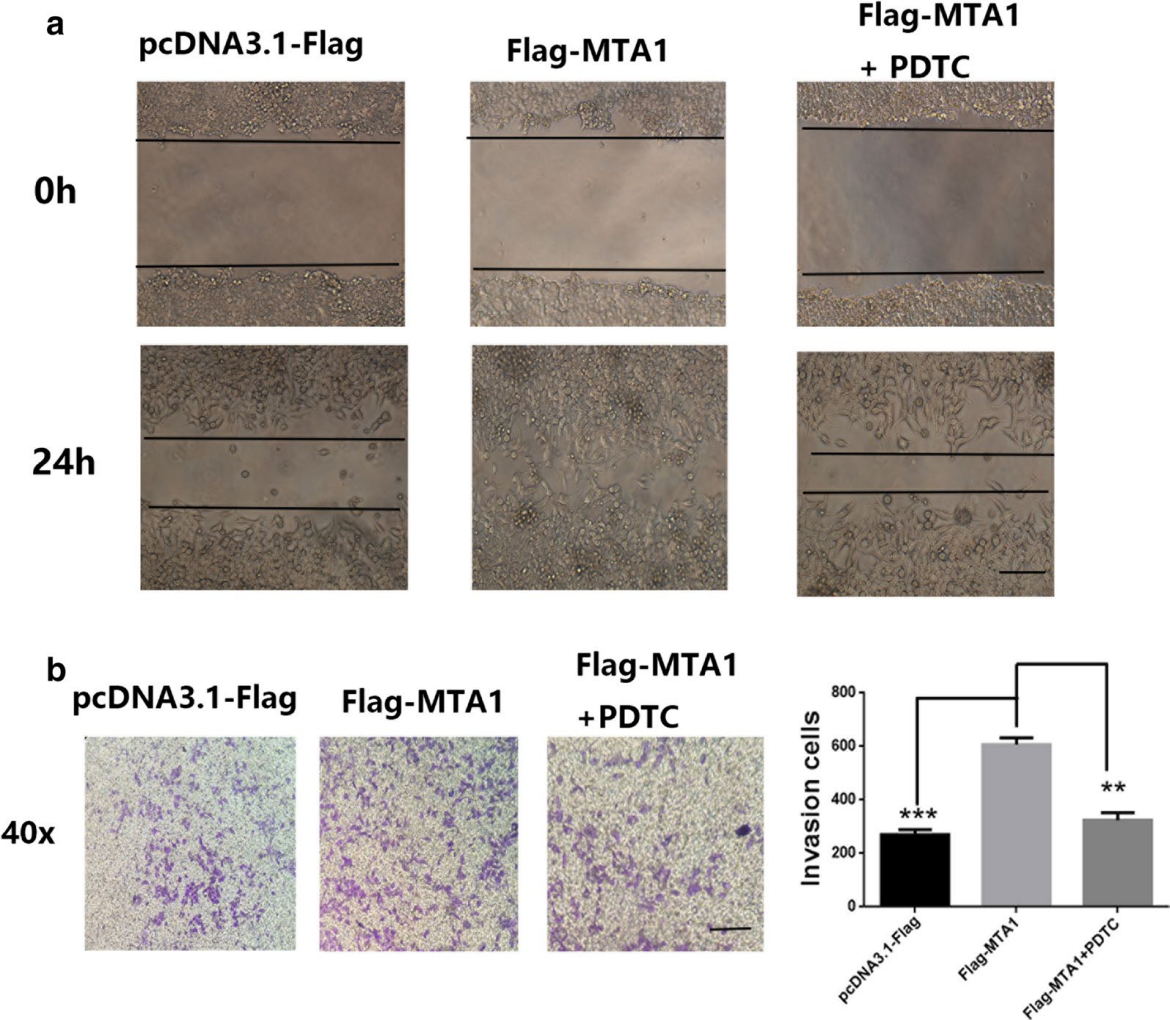
MTA1 regulated the migration and invasion of RCC cells via NF-κB. A498 cells were transfected with pcDNA3.1-Flag, Flag-MTA1, and Flag-MTA1 + PDTC (10 nM). **a** In the wound healing assay, MTA1 improved the migration of RCC cells, while the addition of the inhibitor PDTC blocked the effect of MTA1 on migration. Scale bar: 100×. **b** In the transwell assay, MTA1 markedly induced invasion of A498 cells, but compared to the MTA1 group, the invasion of MTA1 + PDTC A498 cells was lower. **p < 0.01; ***p < 0.001; Scale bar: ×40

We further studied the inhibitory role of PDTC treatment on migration and invasion of RCC cells. We transfected the A498 cells with pcDNA3.1-Flag, Flag-MTA1, and treated Flag-MTA1 with PDTC (10 nM), before performing the wound healing and transwell assays. As shown in Fig. 5a, compared to the Flag-MTA1 group, the invasion of Flag-MTA1 cells treated PDTC was lower. The transwell assay showed a similar result: PDTC treatment blocked the effect of MTA1 on invasion (Fig. 5b). These results suggested that MAT1 may affect invasion and migration through the NF-κB signaling pathway.

## Discussion

MTA1 reportedly up-regulated and promoted the progression of tumor growth in many cancers, including lung, liver, breast, ovarian, gastric, and prostate cancers [7–9, 21, 22]. Nonetheless, the role of MTA1 in RCCs is still unclear. In renal cancers, ccRCC comprises nearly 80% of all RCCs. In this study, we selected 109 pairs of ccRCCs and adjacent normal tissues and demonstrated that the expression level of MTA1 was significantly up-regulated in ccRCC tissues, compared to the surrounding tissue (Fig. 1 and Table 1). Moreover, MTA1 was highly expressed in A498 and 768-O RCC cell lines (Fig. 1).

We found that the overexpression of MTA1 markedly promoted A498 cell migration and invasion in vitro (Figs. 2, 3), which strongly suggests that MTA1 was the key factor regulating the metastasis of RCCs. However, the clinical-pathological analysis revealed that positive MTA1 expression in RCCs was associated with the age of patients and T stage and grade but not with the lymph node metastasis (Table 2). This apparent contradiction could likely be attributed to the small number of cases with lymph node metastasis enrolled in this study, which did not lead to a statistically significant difference. Furthermore, Table 2 reports that 19 MTA1-positive cases were present, while only 4 MTA1-positive cases presenting lymph node metastasis were present. A similar result was shown in the no lymph node metastasis group, that is, MTA1-postive cases were more common than cases with negative MTA1. This trend is consistent with the results in our in vitro experiments. In addition, the enrolled cases were nearly early-stage; thus, over half of the patients in early-stage disease showed MTA1 positivity. Due to the limitation of the metastasis group sample size, future studies with a larger number of cases are needed to further examine the relationship between MTA1 and lymph node metastasis or distant metastasis.

Mechanistically, we showed that MTA1 was able to regulate the metastasis of RCCs by mediating the expression of E-cadherin, MPP2, and MPP9 (Fig. 4). Moreover, we used PDTC to inhibit the NF-κB signaling pathway, which limited the effects of MTA1 (Fig. 4). Further, the in vitro migration and invasion assays in the presence of PDTC treatment showed that NF-κB played a vital role in the MTA1-mediated migration and invasion (Fig. 5). Considering all the above evidence, we concluded that MTA1 mediated the migration and invasion of RCCs by targeting E-cadherin, MPP2, and MPP9 via the NF-κB pathway. Similar to our findings relative to the MTA1-mediated migration and invasion of RCCs by targeting E-cadherin, MPP2, and MPP9, Yao et al. reported that MTA1 promoted cell proliferation and invasion by regulating MMP2 and MMP9 in gastric cancers [13]. MTA1 could have an effect on some signaling pathways, including hedgehog, PI3K, HIF, and AKT [8, 12]. Bui-Nguyen et al. showed that MTA1 was a target gene of NF-κB [18]. In this study, we revealed that the NF-κB signaling pathway was a new downstream target for MTA1, although the mechanism of how MTA1 affects the NF-κB signaling pathway requires further study.

## Conclusions

In summary, our study found that MTA1 is overexpressed in RCC cells and tissues. MTA1 mediated the tumor cell migration and invasion in cultured cells through the regulation of E-cadherin, MPP2, and MPP9 via the NF-κB pathway. These results suggested that MTA1 might play a vital role in the progression of RCC.

## Supplementary information

**Additional file 1.**

## Data Availability

The supporting data of this study are available from the corresponding author upon request.

## Abbreviations

MTA1: Metastasis-associated gene 1
RCC: Renal cell carcinoma
MMP2: Matrix metalloproteinase 2
MMP9: Matrix metalloproteinase 9
