# Supplementary material for: Elevated MTA1 induced the migration and invasion of renal cell carcinoma through the NF-κB pathway
Source: BMC Urol. 2020 Oct 15;20:160. doi: 10.1186/s12894-020-00731-1 (PMC7558699; doi:10.1186/s12894-020-00731-1)
Supplement: Supplementary file 1 — Additional file 1. [file 12894_2020_731_MOESM1_ESM.docx]

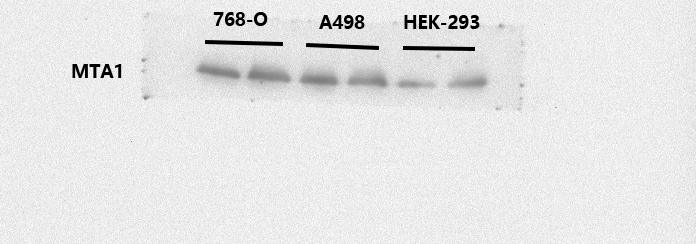


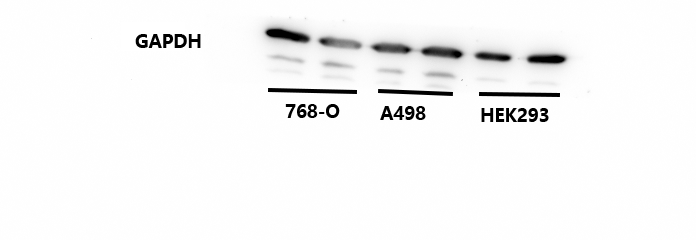


**Additional figure1**

**Original data of figure 1C:** The expression of MTA1 in RCC cell lines. Normal renal cell line HEK293T and RCC cell lines A498 and 768-O cell lysates were used in western blotting analysis with MTA1 and GAPDH antibodies.

**
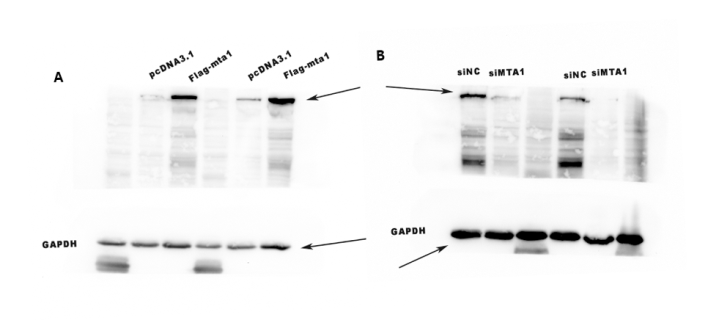
**

**Additional figure2**

**Original data of figure 4A:** Protein levels of MTA1. A498 cells were transfected with pcDNA3.1-Flag, Flag-MTA1, siNC and si-MTA1, and cells were collected for WB with MTA1 and GAPDH antibodies. (A) The bands of over-expressed MTA1 and GAPDH. (B) The bands of down-expressed MTA1 and GAPDH with arrow

**
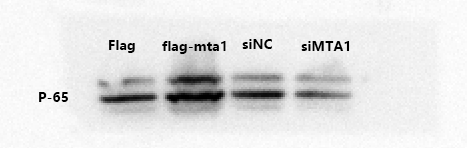

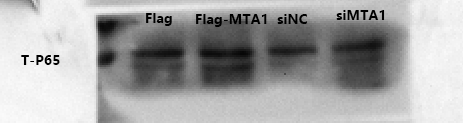

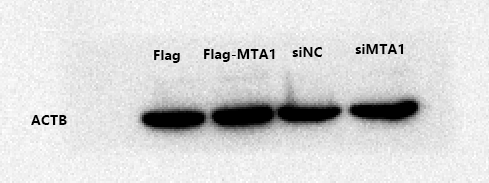
**

**Additional figure3**

**Original data of figure 4E:** A498 cells were transfected with pcDNA3.1-Flag, Flag-MTA1, siNC and si-MTA1. After48 hours, cells were subjected to western blotting analysis using antibodies targeting p-p65, T-p65and ACTB.
